# Human impacts on mammals in and around a protected area before, during, and after COVID‐19 lockdowns

**DOI:** 10.1111/csp2.12743

**Published:** 2022-06-07

**Authors:** Michael Procko, Robin Naidoo, Valerie LeMay, A. Cole Burton

**Affiliations:** ^1^ Department of Forest Resources Management, Forest Sciences Centre University of British Columbia Vancouver British Columbia Canada; ^2^ WWF‐US Washington District of Columbia USA; ^3^ Institute for Resources, Environment and Sustainability University of British Columbia Vancouver British Columbia Canada

**Keywords:** anthropause, coastal forests, mammal conservation, mesopredator release, predator shield, recreation ecology

## Abstract

The dual mandate for many protected areas (PAs) to simultaneously promote recreation and conserve biodiversity may be hampered by negative effects of recreation on wildlife. However, reports of these effects are not consistent, presenting a knowledge gap that hinders evidence‐based decision‐making. We used camera traps to monitor human activity and terrestrial mammals in Golden Ears Provincial Park and the adjacent University of British Columbia Malcolm Knapp Research Forest near Vancouver, Canada, with the objective of discerning relative effects of various forms of recreation on cougars (*Puma concolor*), black bears (*Ursus americanus*), black‐tailed deer (*Odocoileus hemionus*), snowshoe hares (*Lepus americanus*), coyotes (*Canis latrans*), and bobcats (*Lynx rufus*). Additionally, public closures of the study area associated with the COVD‐19 pandemic offered an unprecedented period of human‐exclusion through which to explore these effects. Using Bayesian generalized mixed‐effects models, we detected negative effects of hikers (mean posterior estimate = −0.58, 95% credible interval [CI] −1.09 to −0.12) on weekly bobcat habitat use and negative effects of motorized vehicles (estimate = −0.28, 95% CI −0.61 to −0.05) on weekly black bear habitat use. We also found increased cougar detection rates in the PA during the COVID‐19 closure (estimate = 0.007, 95% CI 0.005 to 0.009), but decreased cougar detection rates (estimate = −0.006, 95% CI −0.009 to −0.003) and increased black‐tailed deer detection rates (estimate = 0.014, 95% CI 0.002 to 0.026) upon reopening of the PA. Our results emphasize that effects of human activity on wildlife habitat use and movement may be species‐ and/or activity‐dependent, and that camera traps can be an invaluable tool for monitoring both wildlife and human activity, collecting data even when public access is barred. Further, we encourage PA managers seeking to promote both biodiversity conservation and recreation to explicitly assess trade‐offs between these two goals in their PAs.

## Graphical abstract

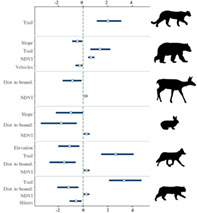

## 1 INTRODUCTION

Worldwide, the creation of protected areas (PAs) is one of the most common methods of conserving biodiversity (Barnes et al., 2017; Sarmento & Berger, 2017), with many PAs regarded as the only remaining barrier keeping hundreds of species from extinction (Pacifici et al., 2020). However, PAs are often established under a dual mandate to conserve biodiversity while also promoting recreational opportunities, presenting a challenge to PA managers, as the two may sometimes be at odds (Sarmento & Berger, 2017; Thomas & Reed, 2019). It is well known that inadequately regulated consumptive recreation (e.g., hunting, trapping, fishing) may pose a threat to biodiversity conservation through the direct removal of wildlife from populations (Schipper et al., 2008). Nonconsumptive recreational activities (e.g., hiking, biking, off‐road vehicles) are often thought to have fewer negative effects on wildlife (Kays et al., 2017), but evidence suggests they may also hamper PA effectiveness by imposing widespread disturbance on the wildlife assemblages inhabiting these spaces (Larson et al., 2016; Reed & Merenlender, 2008).

Effects of nonconsumptive recreation on wildlife may include wildlife avoidance of hikers (Erb et al., 2012) and mountain‐bikers (Naidoo & Burton, 2020), increased physiological stress (Arlettaz et al., 2007), reduced reproductive success (Finney et al., 2005), or increased habituation leading to shifts in predator–prey dynamics (Geffroy et al., 2015). To this end, recreation may lead to predators avoiding areas of higher human influence, while prey species and mesopredators may subsequently select these “predator shields” as a release from top‐down pressures (Berger, 2007; Erb et al., 2012; Prugh et al., 2009; Sarmento & Berger, 2017). Nevertheless, species exposure to recreation may vary with park accessibility by people (Larson et al., 2018) as well as with habitat quality (Reilly et al., 2017), and methods of assessing recreation effects on wildlife are sometimes debated (Bateman & Fleming, 2017) or under development (Marion et al., 2020).

In North America, the rapid and continued growth of the recreation industry (White et al., 2016) reinforces an urgent need to understand how wildlife respond to larger numbers of recreationists in PAs. Recreation is well‐known to promote human health (Willis, 2015; Wolf et al., 2020), cultural resilience (ISRC & CPRA, 2015), and economic prosperity (Naidoo et al., 2016), and increases in recreational activity may be driven by population growth (White et al., 2016), social media (Winter et al., 2019), or prolonged summers caused by climate change (Hewer & Gough, 2018). These increases are commonly seen in national parks, but locally managed parks also see increasing numbers of recreationists, potentially due to greater accessibility (Larson et al., 2018; White et al., 2016), underscoring a need for recreation ecologists to consider how recreation affects wildlife across a range of PAs.

The British Columbia (BC), Canada provincial park system is the largest sub‐national park system in North America, with annual visitation estimated at 25.8 million visitors, and the most heavily used parks each seeing nearly 1 million day‐users (BC Ministry of Environment, 2019a). Over 75% of BC Parks are “dedicated to the preservation of their natural environments for the inspiration, use and enjoyment of the public” (BC Ministry of Environment, 2021), with some seeing visitation increases of over 168% in the last decade (e.g., Joffre Lakes Provincial Park; BC Ministry of Environment, 2019b). Moreover, BC has the highest levels of biodiversity and species at risk in all of Canada, and is particularly important for larger mammal species (Shackelford et al., 2018; Westwood et al., 2019), suggesting PA management and conservation efforts need to be guided by strategic planning and prioritization (Martin et al., 2018). However, such evidence‐based decision‐making may be difficult without understanding how visitation affects wildlife in PAs.

Recent lockdowns associated with the COVID‐19 pandemic, which some have termed the “anthropause” (Rutz et al., 2020), provided an unprecedented “experimental” exclusion of human activity which could help inform how wildlife are impacted by human activity (Bates et al., 2020). Worldwide, biologists hypothesized how the natural world might respond to the anthropause (Corlett et al., 2020; Gaynor et al., 2020; Rutz et al., 2020), with some of the first emerging studies showing increased sightings of elusive species (Silva‐Rodríguez et al., 2020) and species reclaiming spaces that had previously been monopolized by humans (Derryberry et al., 2020; Manenti et al., 2020; Wilmers et al., 2021). Yet, these studies have largely focused on urban areas (e.g., Manenti et al., 2020; Silva‐Rodríguez et al., 2020), exurban residential areas (e.g., Wilmers et al., 2021), or non‐mammalian species (e.g., Derryberry et al., 2020). Due to the recentness of COVID‐19 lockdowns, and the ongoing nature of the pandemic, there has been little evidence regarding how a lack of recreation during the anthropause may have impacted mammals in PAs. Further, data loss during this time was notable due to many researchers being unable to safely continue field research (Pennisi, 2020). This unexpected obstacle highlights the utility of autonomous monitoring devices like camera traps (CTs) (Blount et al., 2021), which offer a cost‐effective and non‐invasive method of collecting continuous data on both human and wildlife activities (Naidoo & Burton, 2020).

Here, we used CTs to investigate the effects of nonconsumptive recreation on wildlife in a PA and adjacent research forest at the doorstep of Canada's most densely populated city—Vancouver, BC (Statistics Canada, 2016). Our study took place within the context of the COVID‐19‐induced anthropause, which mandated a system‐wide shutdown of BC provincial parks for over a month in spring 2020 and a similar shut‐down within the research forest used in this study. Our objectives were to: (1) quantify the amount and types of anthropogenic activity occurring throughout this multiple‐use landscape; (2) test the effects of recreational activities on the habitat use of species inhabiting the area; and (3) investigate how detection rates of each of these species may have changed during and after the anthropause. We hypothesized that larger predatory species such as cougars would be negatively impacted by recreation, implying a form of predator avoidance of human‐derived risk, and that prey species such as black‐tailed deer would therefore be positively associated with recreation, consistent with the predator shield hypothesis (Berger, 2007; Sarmento & Berger, 2017). We also hypothesized that mesopredators such as coyotes would be positively associated with human activity, due to the mesopredator release hypothesis (Erb et al., 2012; Prugh et al., 2009). Specifically, we predicted that while controlling for environmental variation, areas or times of lower human use would see greater predator habitat use, and lower prey and mesopredator habitat use, with areas or times of higher human use seeing opposite trends. As a result of these expectations, we predicted that predator detection rates would increase during the anthropause, while prey and mesopredator detection rates would decrease due to anticipated reductions in human activity during this time.

## 2 MATERIALS AND METHODS

### 2.1 Study area

**FIGURE 1:**
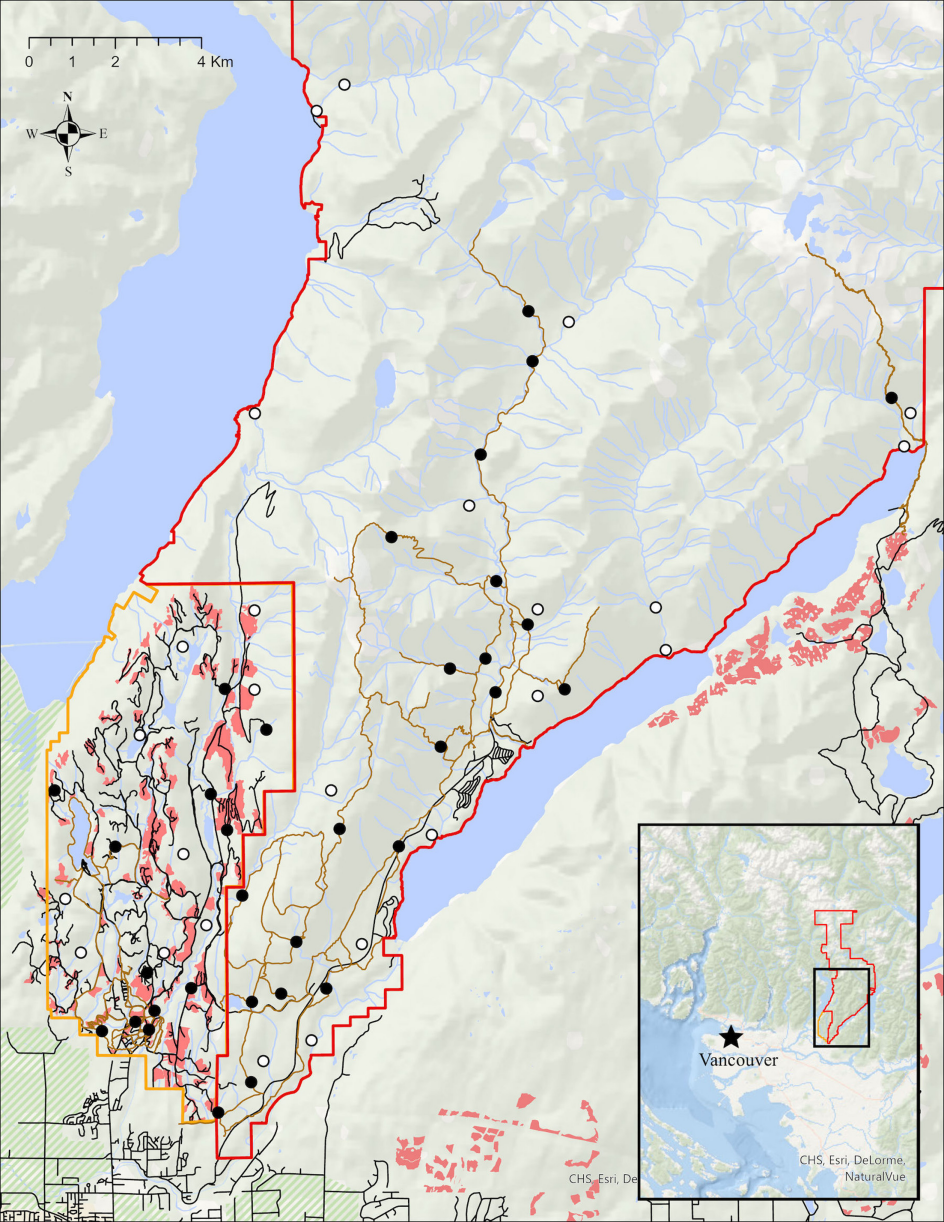
Study area of Golden Ears Provincial Park (red outline) and Malcolm Knapp Research Forest (orange outline) in southwestern British Columbia, Canada. Black circles represent on‐trail camera trap locations, and white circles represent off‐trail camera trap locations. Black lines represent roads, brown lines represent recreational trails, green (hashed) polygons represent agricultural land reserves, red polygons represent harvested forest from the past 5–20 years, and blue polygons/lines represent hydrological features (lakes/streams)

This study was conducted in the adjacent landscapes of Golden Ears Provincial Park (hereafter, “Golden Ears”) and the University of British Columbia (UBC) Malcolm Knapp Research Forest (hereafter, “Malcolm Knapp”) in southwestern BC, Canada (Figure 1). Adjacent to the Greater Vancouver Region, Golden Ears spans approximately 625 km^2^ and supports a range of forest communities, including lower elevation forests with understories of vine maple (*Acer circinatum*) and salmonberry (*Rubus spectaculus*) beneath canopies of western hemlock (*Tsuga heterophylla*), yellow cedar (*Chamaecyparis nootkatensis*), western red cedar (*Thuja plicata*), and Coastal Douglas‐fir (*Pseudotsuga menziesii* var. *menziewii*), and high elevation forests with sparse krummholz trees alongside white mountain‐heather, mosses, and lichens (BC Ministry of Environment, 2013). The park permits a variety of recreational activities such as camping, hiking, mountain biking, horseback riding, fishing and boating, but excludes hunting and off‐road vehicles. Golden Ears also contains the largest campground in the province, receives almost one million recreational visitors annually, and recently reported the most annual campers and the second most annual day‐users of any BC provincial park (BC Ministry of Environment, 2019a). These recreational pressures are the main form of anthropogenic disturbance in the park, as resource extraction has been prohibited since 1929, rendering the park a prime example of a protected coastal forest with high levels of human visitation.

Malcolm Knapp is a 52 km^2^ research forest that abuts the southwestern boundary of Golden Ears and contains a similar forest ecosystem, but has the principal management objectives of providing research and educational opportunities for UBC students and others. Designated as a research forest reserve in 1943, the privately owned forest includes an extensive network of over 100 km of roads, which facilitate the main goals of education and research activities, as well as forestry operations (e.g., harvesting, thinning, fertilization, and replanting). Malcolm Knapp also receives thousands of recreational visitors annually who access approximately 30 km of trails. However, road access is curtailed via a strict gated access route, and camping, hunting, fishing, and off‐road recreation are not permitted in the forest. There are also reservable facilities for recreation and other user groups outside of UBC student groups, but activities associated with these facilities are contained within a small (<2 km^2^) portion of the forest. Roads in Malcolm Knapp can be used for hiking, but motorized usage is restricted only to those with access to the main gate (i.e., Malcolm Knapp staff, UBC researchers, and groups registered for activities on site). Therefore, the main drivers of human disturbance in Malcolm Knapp are related to educational and research activities, including forestry operations, but some areas see mild‐to‐moderate levels of nonmotorized day‐use recreation.

In the Spring of 2020, Golden Ears and Malcolm Knapp were temporarily closed due to the COVID‐19 pandemic. Golden Ears was closed to the public from 8 April to May 13, 2020, after which it opened for limited day‐use (under a permit system) on 14 May and camping on June 1, 2020. Malcolm Knapp was closed from 23 March to June 5, 2020. During these times, public access was heavily restricted, with substantial fines being imposed on individuals who entered these areas without authorization. However, limited education, research, and forest operations in Malcolm Knapp were permitted during the closure.

### 2.2 Camera trap sampling design

Beginning in March 2019, we deployed a stratified random CT array both on and off human‐use trails and roads to monitor medium‐ and large‐bodied mammals, hikers, mountain bikers, horseback riders, and vehicles. Strata in Golden Ears included locations along high human use trails, low human use trails, and off‐trail (cameras placed >250 m and <1 km from trails). High/low use designations of trails were based on local knowledge and expert opinion of park managers (S. Stickney, pers. comm). In Malcolm Knapp, two strata were used: on‐trail (or road) and off‐trail, with the latter similarly having cameras placed >250 m and <1 km from both trails and roads. We used a buffer of 250 m to 1 km due to difficulty accessing off‐trail locations in the steep park terrain. Cameras were separated by a minimum of 300 m.

We navigated as close as feasible to each random point and attached a Reconyx Hyperfire Pro 2 (Reconyx, Holmen, WI, USA) CT to a nearby tree. CTs were set for a target detection zone, focused on either a recreation trail or road (“on‐trail”), or a game trail (“off‐trail”) approximately 3–5 m in front of the lens. CTs were set at approximately 1 m in height to maximize detection probability of medium‐ and large‐bodied wildlife species, while also restricting the amount of identifiable human features captured in photos to protect the privacy of individuals using the landscape. We took care to ensure camera heights and distances to target zones were similar across the study, as small changes in these can drastically impact detectability (Miller et al., 2017), thereby biasing results.

The final array consisted of *n* = 58 camera “stations,” including 37 in Golden Ears (20 on‐trail, 17 off) and 21 in Malcolm Knapp (13 on‐trail or road, and 8 off). Most stations were active for at least 1 year, with some periods of camera inactivity due to malfunctions, vandalism, and theft (Appendix S1), but periodic SD card collections were performed to minimize these until final collections in August–September 2020.

Photos containing humans were blurred using a facial redaction software (Lixar, 2018) to ensure privacy protection. These images were then uploaded to a custom camera trapping database for identification (WildCo, 2021), which utilized an automated detection software to sort the photos into human, vehicle, and animal categories, before subsequent review by the project team (Fennell et al., 2022). We identified species for all images containing mammalian wildlife and domestic dogs, and we classified images of humans into the following categories: hikers, mountain bikers, horseback riders, and motorized vehicles. Data were then imported to R (version 3.6.2) for data management and statistical analyses (R Core team, 2019).

### 2.3 Analytical framework

Prior to modeling, we defined independent detections of wildlife as images of the same species taken at least 30 min apart at each station (Burton et al., 2015). Independent detections of human activity were determined using an independence threshold of 1 min, since numerous recreationists were likely to use high‐traffic trails within the span of 30 min. We therefore assumed that multiple wildlife photos taken within 30 min of each other contained the same individuals or groups, while multiple human photos taken more than 1 min after another would contain different individuals or groups, given most recreationists travel unidirectionally along trails. We selected focal wildlife species for modeling using three criteria: (1) those with at least 30 independent detections; (2) those with at least medium body size (>1 kg); and (3) those with primarily terrestrial rather than arboreal movement. Our response variable was a binary detection/non‐detection for each focal species during each week at each station. We chose this “station‐week” temporal scale to assess temporal variation in species habitat use in response to human activity, while avoiding an excess of zeros in detection histories at finer temporal scales (e.g., “station‐days”; Naidoo & Burton, 2020).

To investigate how recreational activities impacted wildlife habitat use, we modeled the “probability of use” (Kays et al., 2021) for each of these focal species during a station‐week with a Bayesian generalized linear mixed‐effects model (GLMM), assuming a binomial response distribution and including station as a random intercept to account for non‐independence among weeks sampled at the same station. Using this GLMM specification, the probability of use of a station during each week for each species was modeled as a function of several hypothesized explanatory variables (Table 1). Since our primary focus was on species responses to recreational activities, we investigated possible effects of hikers, mounted recreationists (i.e., horseback riders and mountain bikers), and motorized vehicles during a given station‐week by including the average weekly detection rates (i.e., number of detections at a station‐week divided by the number of days the camera was active during that week, typically 7) as possible explanatory variables.

Eleven additional variables were included to control for alternative hypothesized drivers of wildlife habitat use variability. These included forest measures, namely, crown closure (%), forest height (m), the percent of forest recently (within 5–20 years) harvested within a 500 m buffer of the station (%), and topographical measures, specifically elevation (m) and slope (angle in °). We controlled for seasonal variation in vegetation productivity using the normalized difference vegetation index (NDVI) calculated using MODIS product VNP13A1 (8‐day interval data at 500 m resolution). We also included distance to nearest water source (m) as a general habitat measure, and distance to the urban‐wildland boundary (m) as an index of the potential influence of residential and agricultural areas surrounding the PA. Finally, we included variables describing variation in deployment across stations that could affect detectability (Hofmeester et al., 2018), namely the height of the camera above the intended target path (m above the recreation trail, road, or game trail), the distance to the intended target path (m), and whether the camera was placed along a road or trail (binary on/off). We therefore assumed that the potential influence of variation in detectability was minimized by our standardized protocols and inclusion of these sampling variables. We did not use an occupancy modeling approach to estimate detectability, as we were interested in variation in detections as a signal of local habitat use rather than as detection error, and we considered it unlikely that the assumptions of occupancy modeling would be met in our sampling context (e.g., site closure; Neilson et al., 2018; Kays et al., 2021). We assumed any temporal autocorrelation was accounted for through the inclusion of the random effect of camera station, and tested for spatial autocorrelation among stations using Mantel tests on model residuals.

To test whether the anthropause impacted focal species, we built three additional Bayesian models for each species utilizing data from: (1) the whole study area; (2) only Golden Ears; and (3) only Malcolm Knapp. We included all dates during the closure, but only pre‐closure (2019) data matching the sample dates for the post‐closure (2020) period (early‐May to mid‐September) were included, to reduce the possibility of any additional weight toward pre‐closure trends given the longer pre‐closure sampling period. We also calculated detection rates (i.e., the number of detections at a station divided by number of days the station was active rather than raw detections) to account for the different sampling effort within the shorter period of the anthropause relative to the longer pre‐ and post‐closure periods. Each model predicted the detection rate of a species before, during, and after the closure time periods using a factor variable to alter the intercept by time period. We used the closure period as the baseline intercept, and tested whether wildlife detection rates before or after the closure differed from the closure period. We did not include other explanatory variables, such as habitat type, as these were consistent for each station throughout the three comparison periods given stations were not moved and time periods represented similar seasons.

**TABLE 1:** Predictor variables used in the Bayesian generalized linear mixed‐effects models, which contrasted the probability of weekly habitat use for focal species against measures of various forms of human activity while controlling for alternative sources of variation. The mean, minimum (min), and maximum (max) of values (on the raw scale) are provided for the 58 camera stations

Variable	Description	Acquisition	Hypothesis	Mean	Min	Max
Hikers	Weekly detection rate per station‐week (# hikers detected per week/# days the camera was active that week)	CT^a^	Negative impact on top predators, positive impact on prey and mesopredators (predator shield & mesopredator release)	4.68	0.00	191.50
Mounted recreation	Weekly detection rate per station‐week (# mountain bikers detected per week/# days the camera was active that week)	CT	Negative impact on top predators, positive impact on prey and mesopredators (predator shield & mesopredator release)	0.07	0.00	6.00
Motorized vehicles	Weekly detection rate per station‐week (# vehicles detected per week/# days the camera was active that week)	CT	Negative impact on top predators, positive impact on prey and mesopredators (predator shield & mesopredator release)	0.04	0.00	9.29
Crown closure^b^	% crown closure at site	GIS^c^	Control for habitat type	64.47	10.00	85.00
Stand height^b^	Projected height of forest at site (m)	GIS	Control for habitat type	33.03	12.10	53.30
NDVI^d^	Normalized difference vegetation index in a week at site, measured in 8‐day intervals (500 m resolution)	GIS	Control for seasonality	0.75	−0.35	0.99
Pct. Harvested	% of recently (between 2000 and 2015) harvested forest within a 500 m buffer around each station	GIS	Control for habitat type and resource availability	0.06	0.00	0.37
Distance to water^e^	Distance to the nearest stream, river, or lake from site (m)	GIS	Control for resource availability	152.94	3.90	744.17
Distance to south boundary^f^	Distance to the nearest urban‐wildlands boundary (m)	GIS	Control for influence of residential areas adjacent to PA	4466.29	39.97	17,008.14
Elevation^g^	Elevation at site (m) (25 m resolution)	GIS	Control for topography	338.29	11.00	1150.00
Slope^g^	Slope at site (degrees) (25 m resolution)	GIS	Control for topography	13.38	1.28	39.92
Trail	Binary indication of whether site was on‐trail/road or off	Field^h^	Control for camera set	0.58	0.00	1.00
Camera height	Height (m) the camera was position at each site	Field	Control for camera set	0.76	0.41	1.24
Distance to target	Distance from the camera lens to the anticipated path of the target (either the center of the trail/road or the nearest game trail) (m)	Field	Control for camera set	3.46	1.32	7.54

^a^ CT acquisition method—data were collected by camera trap.

^b^ Data Source: Data Management and Access—Province of British Columbia.

^c^ GIS acquisition method—data were collected using geoprocessing tools in ArcGIS Pro.

^d^ Data Source: MODIS Satellite Product VNP13A1.

^e^ Data Source: Freshwater Atlas—Province of British Columbia.

^f^ Data Source: Shapefiles of boundaries provided by BC Parks and Malcolm Knapp Research Forest Management.

^g^ Data Source: Digital Elevation Model—Province of British Columbia.

^h^ Field acquisition method—data were collected in the field.

For the models used to examine recreational activity effects on probability of habitat use, we tested for excessive multicollinearity that would cause parameter estimation instability using pairwise correlations (all *r* < 0.7; Zuur et al., 2010) (Appendix S2). We used Kendall's tau and Spearman's rho for any correlations with the binomial variable, Trail, and Pearson's correlation for all other pairs. We normalized all continuous explanatory variables by subtracting the mean and dividing by the standard deviation (SD) to facilitate comparisons of relative effects of variables on the probability of species habitat use. These models were constructed using flat priors, and run with four chains of 100,000 iterations (burn‐in period of 50,000) to ensure model convergence. Models to investigate changes in detection rates between time periods were constructed with flat priors, and run with four shorter chains of 10,000 iterations (burn‐in period of 2000), which were sufficient for model convergence. For all models, convergence of posterior distributions was confirmed visually with trace plots and with the Gelman‐Rubin statistic (R‐hat) (Hobbs & Hooten, 2015). Variables were considered to have strong evidence for effects on wildlife detections if 0 was not within their 95% credible parameter estimate intervals. Models were implemented using the R package *brms* (Burkner, 2017).

## 3 RESULTS

### 3.1 Summary of survey

From the 58 CTs deployed across Golden Ears and Malcolm Knapp, we collected and classified 1,059,703 images from 23,928 camera trap‐days of sampling effort, which provided detection data for 3479 station‐weeks used in modeling. We detected 19 mammalian wildlife species (Appendix S3), six of which met our criteria for modeling, namely, black‐tailed deer (*Odocoileus hemionus*, *n* = 709 independent detections using 30 min. threshold), coyote (*Canis latrans*, *n* = 416), black bear (*Ursus americanus*, *n* = 290), snowshoe hare (*Lepus americanus*, *n* = 248), bobcat (*Lynx rufus*, *n* = 203), and cougar (*Puma concolor*, *n* = 46). Black bears were the most widely distributed of these wildlife species, being detected at 44 of our 58 stations (76%), whereas snowshoe hares showed the most restricted distribution of our six focal species, being detected at only 26 (45%) of all stations (Appendix S3). Notable detections of species that were too infrequently detected to model included the possibly threatened but data deficient western spotted skunk (*Spilogale gracilis*, *n* = 7) and one individual from a recently (c. 2007–2008) reintroduced elk population (Roosevelt subspecies; *Cervus canadensis roosevelti*, *n* = 1).

**FIGURE 2:**
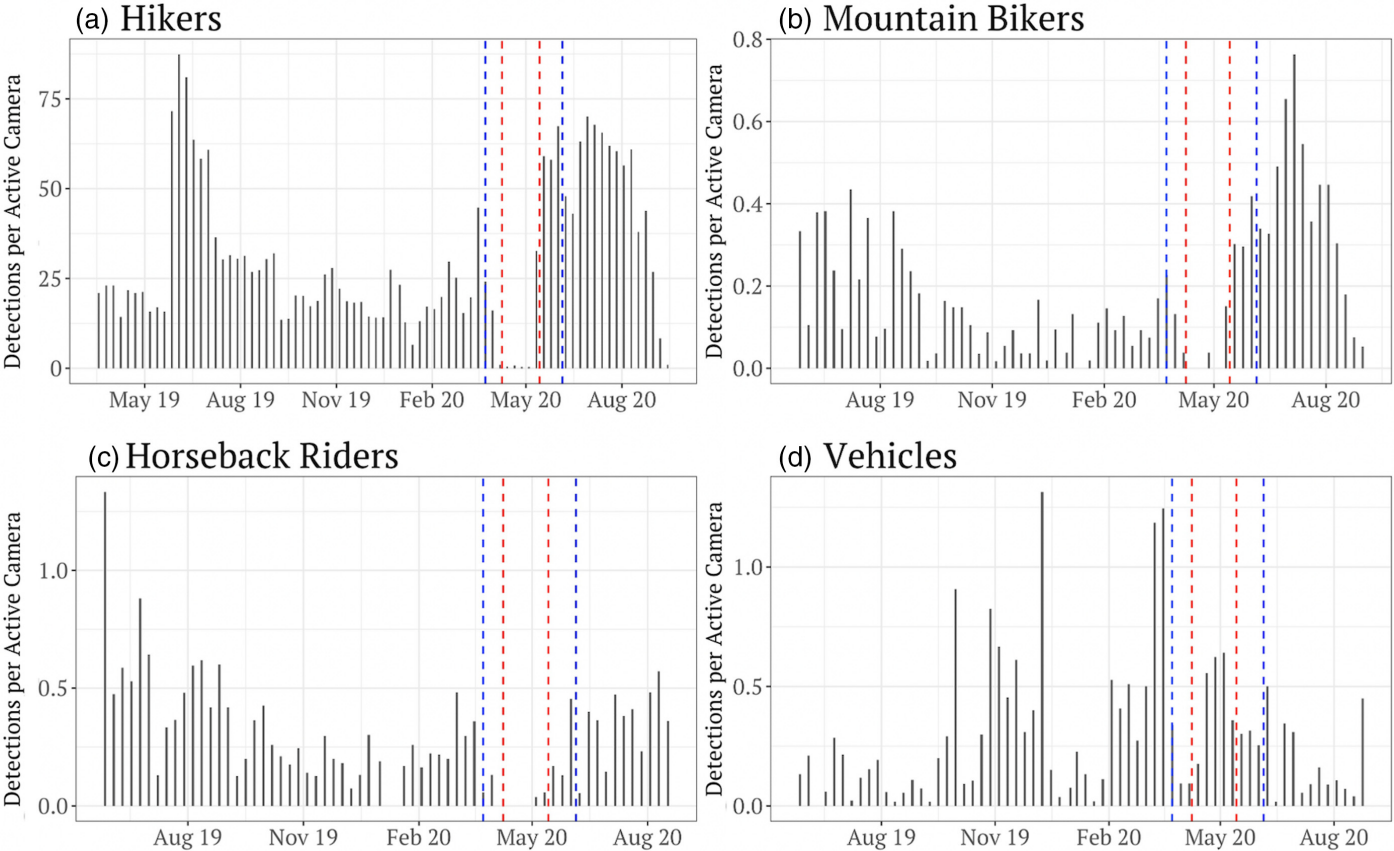
Weekly detections per active camera of (a) hikers, (b) mountain bikers, (c) horseback riders, and (d) motorized vehicles. Horizontal axes represent time throughout the year (in weeks), and vertical axes represent the number of detections per active camera (standardized rate to account for differences in sampling effort among weeks). Spaces between the red dashed lines indicate the COVID‐19‐related closure periods of Golden ears Provincial Park, and spaces between the blue dashed lines indicate the COVID‐19‐related closure periods of Malcolm Knapp Research Forest

Our camera‐trap sampling also collected over 111,486 independent detections of humans (1 min. threshold). Detected activities included 108,865 hiker detections, 1584 mounted recreationists, and 1037 vehicles, with a majority of hikers (*n* = 104,325) and all but one mounted recreationist (*n* = 1583) being detected in Golden Ears, and nearly all vehicles (*n* = 946) being detected on roads in Malcolm Knapp. Although we also identified 31,562 detections of domestic dogs, these were highly correlated with hikers (*r* = 0.92) and therefore not used as a separate predictor variable in models. Park closures resulting from the COVID‐19 pandemic were very effective, leading to reduced recreationist detections during these times (Figure 2). However, working operations (e.g., park maintenance, forest harvest) were detected during the closure at comparable numbers to pre‐ or post‐closure (Figure 2).

### 3.2 Drivers of weekly wildlife habitat use

**FIGURE 3:**
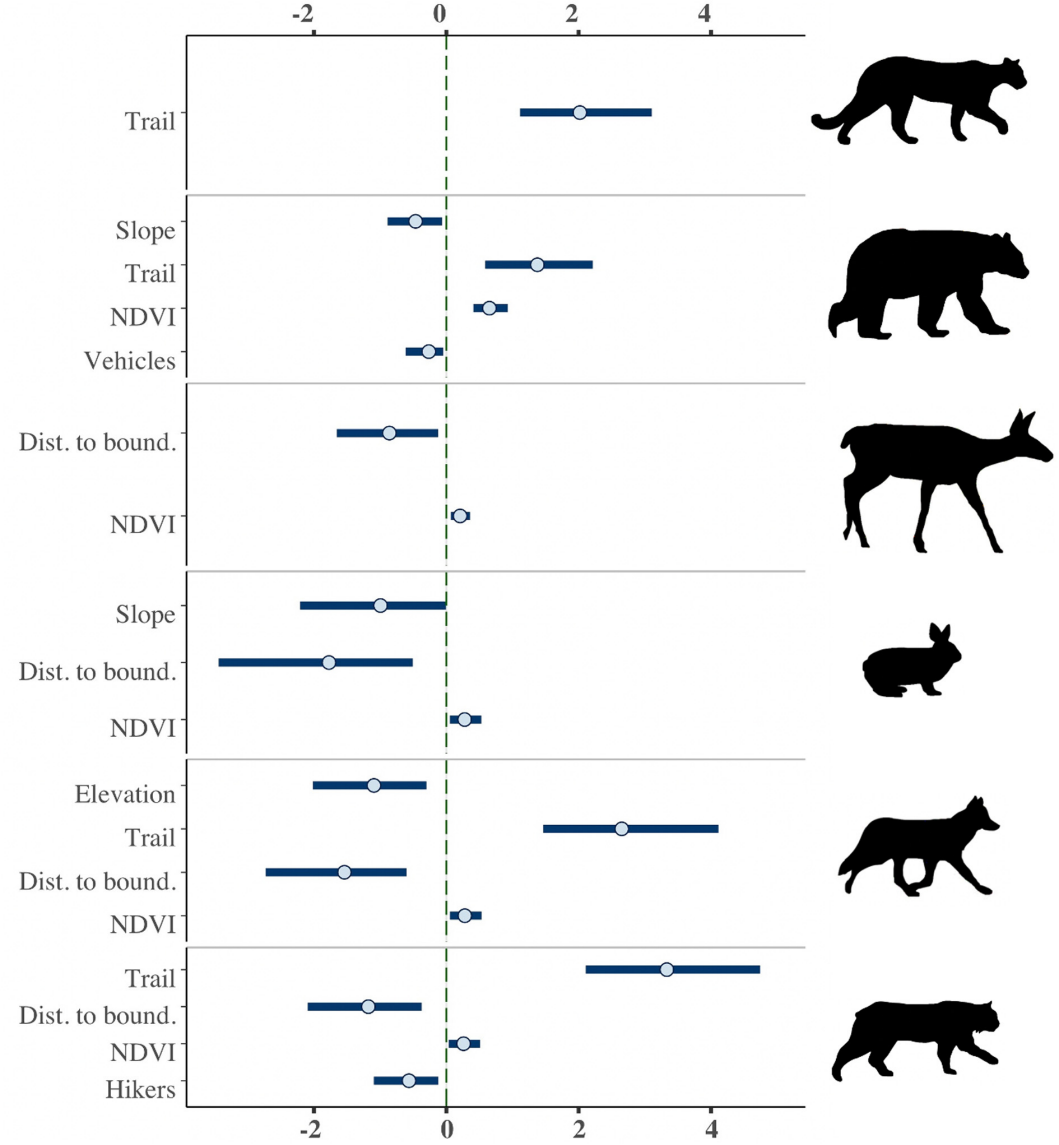
Predictor variables (*y*‐axis) which were strongly associated with species probability of habitat use in Bayesian generalized linear models contrasting species probability of habitat use against a suite of explanatory variables. Focal species (from top to bottom) included cougar, black bear, black‐tailed deer, snowshoe hare, coyote, and bobcat. The horizontal (*x*) axis illustrates posterior distributions, with points in each line representing mean posterior estimates and blue lines representing 95% credible intervals. The hashed green line is the *x*‐intercept of 0, which was used to determine strength of predictor variables (predictor variables had strong evidence if 95% credible intervals of posterior estimates did not overlap zero)

Of the habitat use models run for the six focal species, two showed strong evidence for associations between wildlife and human activity (all models successfully converged with R‐hat <1.1 for all model terms; Appendix S4). Specifically, bobcat habitat use was negatively associated with hikers (mean posterior estimate = −0.58, 95% credible interval [CI] −1.09 to −0.12; Figure 3) and black bears were negatively associated with motorized vehicles (estimate = −0.28, 95% CI −0.61 to −0.05; Figure 3). No other species showed strong evidence of associations with direct measures of human activity. However, several other predictor variables included to control for variation showed strong evidence of impacting species' probability of habitat use (Figure 3). We identified strong evidence that the distance from each station to the urban‐wildland boundary influenced the probability of habitat use for four of six species (all except black bears and cougars), with black‐tailed deer, snowshoe hares, coyotes, and bobcats all being more likely to be detected at stations closer to the boundary (estimates & 95% CIs: black‐tailed deer = −0.87, −1.65 to −0.12, snowshoe hares = −1.82, −3.44 to −0.51, coyotes = −1.57, −2.73 to −0.60, bobcats = −1.19, −2.09 to −0.37). Whether the camera was situated along a trail or road was positively associated with probability of habitat use for all predatory species (estimate & 95% CI: cougars = 2.04, 1.11 to 3.10, black bears = 1.38, 0.59 to 2.21, coyotes = 2.69, 1.46 to 4.11, bobcats = 3.36, 2.11 to 4.74). Further, environmental variation indicated by variables such as elevation, slope, and NDVI also showed strong evidence of impacting species' habitat use (Figure 3).

### 3.3 Wildlife detection rates in the Anthropause

**FIGURE 4:**
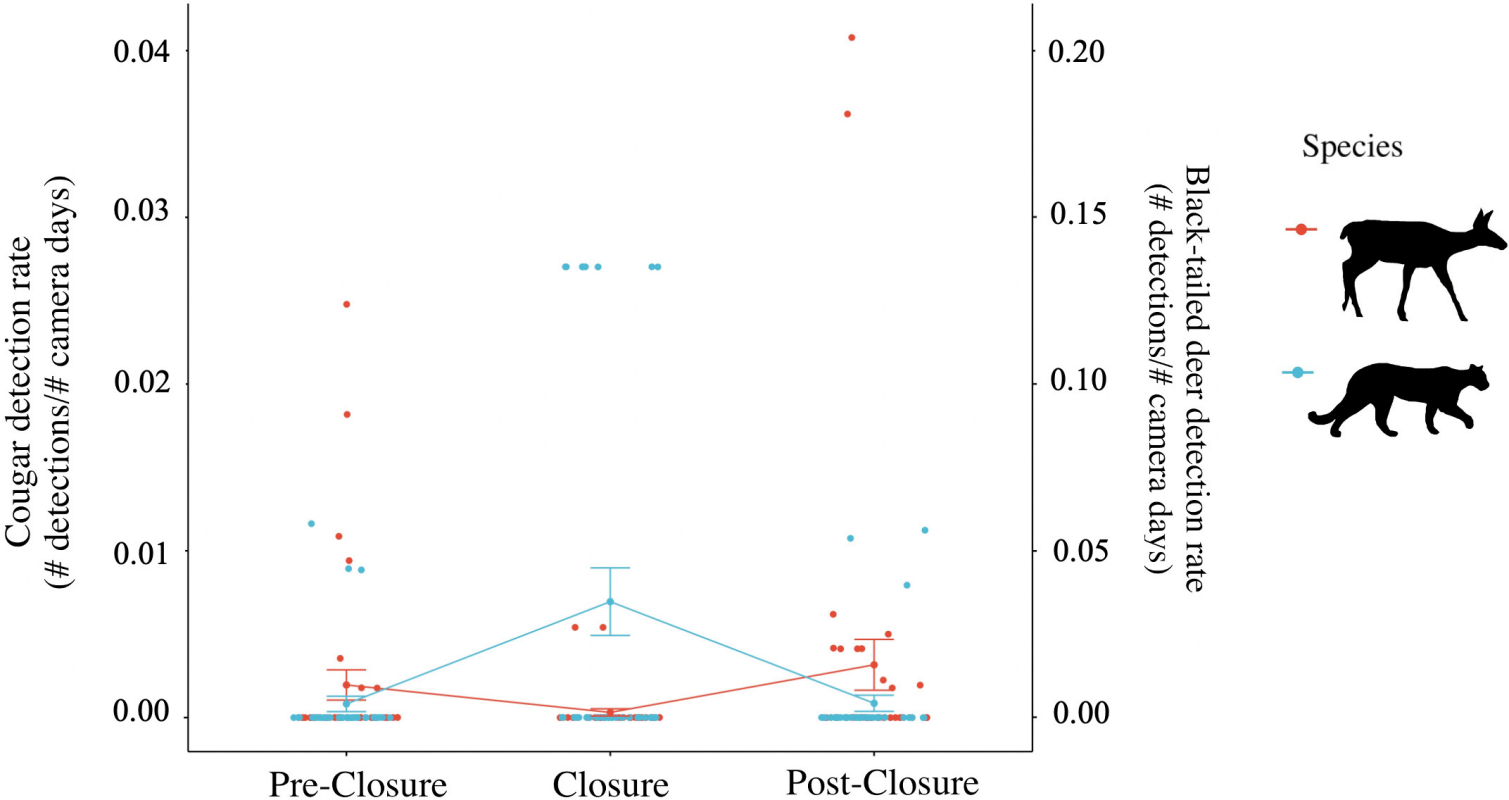
Detection rates (detections/camera days; y‐axes) per station, with data restricted to Golden Ears, before, during, and after the COVID‐19‐related closures (*x*‐axis), for cougars (blue dots and lines; left *y*‐axis) and black‐tailed deer (red dots and lines; right *y*‐axis). Each dot represents the detection rate for a single camera during a given period, with connecting lines illustrating changes between periods. Also shown are the means with whiskers showing the standard errors around these means

From models constructed with data from the entire study area, cougars were the only species to show greater detection rates during the anthropause (estimate = 0.005, 95% CI 0.004 to 0.007, Appendix S5) than before or after the anthropause, with the intercept of the detection model dropping by −0.005 for before (estimate = −0.005, 95% CI −0.007 to −0.003) or by −0.004 after (estimate = −0.004, 95% CI −0.006 to −0.002) relative to during the anthropause. When data were restricted to Golden Ears, cougars were similarly detected at a higher rate during the closure (estimate = 0.007, 95% CI 0.005 to 0.009) than before or after the closure (both estimates = −0.006, both 95% CIs −0.009 to −0.003), while black‐tailed deer showed higher detection rates after the closure (estimate = 0.014, 95% CI 0.002 to 0.026) than during (estimate = 0.002, 95% CI −0.007 to 0.010), but no difference was found between during and before (Figure 4, Appendix S5). In Malcolm Knapp, there was no strong evidence of changes in species detection rates across time periods (Appendix S5).

## 4 DISCUSSION

Our findings provide evidence of shifts in wildlife habitat use in response to variation in recreational pressure at the weekly scale, as well as dramatic changes in recreational activity as a result of the closures imposed by the COVID‐19 pandemic, coinciding with some shifts in species' detection rates. We predicted that larger predators would avoid areas and times of higher recreation, allowing prey and mesopredator species to evade predation pressure by positively associating with recreation. These predictions were not supported by the data, with mesopredator bobcats being negatively impacted by hikers and large predator black bears being negatively impacted by motorized vehicles. Negative relationships between bobcats and hikers have been observed elsewhere (George & Crooks, 2006; Reed & Merenlender, 2008). Therefore, while this finding does not support the mesopredator release hypothesis, it provides additional support for concerns that high levels of recreation may impact bobcat habitat use. Additionally, the strong negative relationship between black bear detections and motorized vehicles is interesting in the context of this study area since most vehicle detections were tied to Malcolm Knapp research and education activities, as well as timber harvesting to facilitate these activities. This implies that although black bears are well‐known to select for regenerating stands following forest harvest (Brodeur et al., 2008), they likely avoid areas with very high human activity, particularly where harvest and other forest management interventions are taking place. Prior research has confirmed that bears tend to avoid spaces or times of higher motorized vehicle activity (Ladle et al., 2018; Naidoo & Burton, 2020), but these results typically pertain to grizzly bears (*Ursus arctos*). Others have shown black bears avoid roads (Carter et al., 2010; Zeller et al., 2019), but these do not measure motorized disturbance directly, and instead use the presence of roads as a proxy for motorized disturbance. Research regarding changes in black bear habitat use in response to direct measures of vehicle activity is sparse given the difficulty in collecting data on both. Thus, we present a novel account of black bear habitat use being negatively impacted by explicit measurements of vehicle use.

Alternatively, the negative association between the distance to the urban‐wildland boundary and habitat use of all prey and mesopredator species may provide a different form of support for the predator shield and mesopredator release hypotheses, given human activity and footprint can differentially impact wildlife (Nickel et al., 2020). Accordingly, prey and mesopredator species may use residential areas near the PA as a shield from larger predatory species. Cougars are known to avoid housing densities above certain limits (Smith et al., 2019), and although cougars commonly traverse urban‐wildland gradients (Alldredge et al., 2019), cougar mortality is often greater in more developed areas (Moss et al., 2015). Likewise, black bears commonly associate with exurban spaces due to anthropogenic resource availability, but face a similar risk of mortality (Braunstein et al., 2020; Laufenberg et al., 2018), which could potentially provide a shield for mesopredator or prey species. Therefore, we suggest future research could target a gradient from exurban residential neighborhoods to PAs to investigate whether larger predators are less likely to use spaces adjacent to PAs, and whether prey or mesopredators more commonly inhabit these areas to avoid predators.

We also found strong evidence that cameras situated along trails or roads were more likely to detect predator and mesopredator species. Prior work has identified preferences for linear features in several mammalian species (Fisher & Burton, 2018), even in regions with strong recreational pressures (Reilly et al., 2017). There is thus potential for animal preference for trails to obscure responses to human activities. However, our inclusion of off‐trail (i.e., game trail) samples and the corresponding binary model covariate controlled for variation in detections due to on‐ or off‐trail placement of CTs. Nevertheless, we recommend future research with more extensive off‐trail sampling (e.g., proportional to available off‐trail habitat) to further evaluate potential confounds between animal trail use and responses to recreation. Additionally, alternative analytical approaches could be used to assess potential finer‐scaled trade‐offs for species that use trails but avoid human activities (e.g., attraction‐avoidance ratios; Naidoo & Burton, 2020).

Our CT sampling provided a unique opportunity to investigate how wildlife responded to the anthropause when the study area was closed to the public. Both the strong increase in cougar detection rates during the anthropause and the strong increase in black‐tailed deer detection rates following the reopening of Golden Ears provide support for the predator shield hypothesis in the context of recreation. Cougars are known to exhibit fear‐responses in response to human voices (Smith et al., 2017; Suraci et al., 2019), and have elsewhere responded positively to the anthropause, expanding their ranges into previously unoccupied areas during this time (Wilmers et al., 2021). This immediate response from a top predator to reductions in human activity has implications for carnivore–human coexistence in shared landscapes, underscoring the ability of carnivores to quickly adapt to human activity (Carter & Linnell, 2016). Additionally, increases in black‐tailed deer detections during the post‐closure period may have been related to the implementation of a day‐use pass system which limited recreation upon the reopening of Golden Ears. This implies the possibility of a threshold in recreation effects, whereby intermediate levels may provide a predator shield by displacing cougars but not deer, while higher levels may displace both predators and prey. Identifying such thresholds of disturbance is integral to providing science‐based recommendations to PA managers (Dertien et al., 2021). Thus, our findings provide support for the use of continuous measures of recreation intensity to inform trail limits. We recommend that future research consider analytical methods which could be used to assess thresholds of recreational disturbance (e.g., nonlinear models: Pinheiro & Bates, 2000; piecewise regression: Malo et al., 2011, Toms & Lesperance, 2003; additive models: Zuur et al., 2009), and thus identify recreation “carrying capacities” to inform park management.

The differences in deer responses to recreation that we observed at shorter (weekly) vs. longer (park closure) periods emphasizes the importance of considering temporal scale in wildlife research. Human–wildlife coexistence can occur across different scales, with some species facilitating coexistence by segregating from people at coarser temporal scales (e.g., weekly, Naidoo & Burton, 2020), while other species may avoid humans at finer scales (e.g., daily, Patten & Burger, 2018, or even hourly, Carter et al., 2012; Nickel et al., 2020). The weekly temporal scale of our first set of models did not consider potential finer‐scale segregation, which may have provided more information regarding wildlife displacement by human activity. However, given the lack of previous monitoring in this landscape, we sought to investigate broader patterns of species composition and distribution, while understanding how these metrics were associated with coarse‐scale anthropogenic (e.g., weekly hiker traffic) or environmental factors (e.g., NDVI). Likewise, our second set of models also made coarse‐scale comparisons, summarizing how wildlife detection rates changed throughout time periods of multiple weeks. However, given the limited closure period and our goal of understanding how wildlife activity differed between the closure and periods when the study area was open, we contend that investigation at this temporal scale was most appropriate. Future work may harness analytical methods better suited to investigate whether wildlife segregate from recreationists at daily temporal scales, or whether even finer‐scale behavioral shifts may facilitate human‐wildlife coexistence in this system (e.g., altered diel activity patterns, Carter et al., 2012).

## 5 CONCLUSIONS

The contrast between a heavily visited protected area and a less recreated research forest with limited public road access provided a unique context in which to assess the effects of anthropogenic disturbance on mammalian habitat use. This, in addition to the restriction of public access for a number of weeks during the anthropause offered a rare unplanned experiment through which to explore effects of human activity on wildlife. We aimed to test both the predator shield and mesopredator release hypotheses in the context of recreational activity, finding little support for either hypothesis at the weekly scale, but stronger support for the predator shield hypothesis when considering how detection rates of focal species varied during and after the anthropause. Specifically, at the weekly scale, our findings indicated that recreation in PAs may indeed hamper some wildlife habitat use, with species such as bobcats being displaced by larger quantities of hikers. Moreover, forest operations and other activities in landscapes adjacent to PAs may also negatively impact black bears, resulting in a mosaic of disturbances for the species. We also found increases in cougar detection rates during the anthropause, with the subsequent reopening of the PA coinciding with decreased cougar detection rates and increased black‐tailed deer detection rates. Thus, our study emphasizes how PAs faced with a challenge to simultaneously conserve wildlife while promoting recreation may benefit from considering species‐ and context‐specific approaches to wildlife management, with a particular emphasis on the most sensitive species. We also underscore the utility of CTs in continuously monitoring recreation and wildlife in PAs both generally, and through periods when public access to these spaces is restricted. In light of the dual mandate for many PAs to provide opportunities for recreation while simultaneously conserving wildlife, we encourage PA managers worldwide to adopt tools (e.g., CTs, trail counters) and strategies (e.g., collaboration with external groups) which could better inform on trends in recreation and wildlife habitat use in order to understand whether the two are at odds in their own systems. While there are undoubtedly financial and logistical constraints to detailed investigations of these trends in many PAs, we suggest that targeted monitoring, research partnerships, and cost‐effective protocols can help overcome current data deficiencies.

## AUTHOR CONTRIBUTIONS

A. Cole Burton conceived the study. Michael Procko and A. Cole Burton conducted the field work. Michael Procko conducted the data analysis. Michael Procko, Robin Naidoo, Valerie LeMay, and A. Cole Burton wrote the paper. A. Cole Burton acquired funding to support the study.

## CONFLICT OF INTEREST

The authors declare no conflicts of interest.

## ETHICS STATEMENT

This research was conducted under protocol A18‐0234 from the University of British Columbia's Animal Care Committee and under protocol H21‐01424 from the University of British Columbia's Behavioral Research Ethics Board.

## Supporting information


**APPENDIX S1** Camera activity plot illustrating each individual camera station's periods of activity and inactivity. The *x*‐axis shows time throughout the entire monitoring period, while the *y*‐axis indicates specific camera stations. Lines indicate periods where each camera was active, and gaps indicate periods of inactivity

**APPENDIX S2** Correlations for pairs of predictor variables. Variables are contrasted against each other to illustrate (a) Pearson's rho for all continuous variables, (b) Kendall's tau and (c) Spearman's rho for all variables, but particularly the binomial variable, Trail. All variables had absolute values of correlations < = 0.7. Abbreviated variables are as follows: “Trail” = binary indication (0, 1) of whether the camera was situated along a trail/road (1) or not (0), “Mounted Rec.” = Mounted Recreationists (detection rate), “Stand Ht.” = Stand height (m), “Dist. to Water” = Distance to water (m), “Dist. to bound” = Distance to the urban‐wildland boundary (m), “Pct. Harvested” = Percent of forest harvest (%) in a 500 m buffer around the camera station, “Cam. Ht.” = Camera height (m), “Dist. to Target” = Distance from the camera lens to the expected path of the target (trail, road, or game trail) (m), and “NDVI” = Normalized difference vegetation index extracted from MODIS satellite at 500 m, 8‐day resolution

**APPENDIX S3** (a) Number of independent detections (*x*‐axis) of each species (*y*‐axis) based on a 30‐min independence threshold for all mammalian wildlife species detected throughout the study. (b) Proportion of stations (*x*‐axis) at which each species (*y*‐axis) was detected throughout the study

**APPENDIX S4** Full results from Bayesian generalized linear models for wildlife probability of weekly habitat use against various measures of human activity, while controlling for alternative sources of variation. Bold text indicates predictor variables which had strong effects on weekly wildlife habitat use

**APPENDIX S5** Results of Bayesian regression models for wildlife detection rates against a categorical designation of time periods before, during, and after the COVID‐19 closures. The baseline intercept was set as the closure period to observe differences between the closure period and before/after. Periods which had strong effects on wildlife detection rates are indicated in bold

## Data Availability

Data and materials are available in a publicly accessible repository: https://doi.org/10.5061/dryad.hx3ffbggk. Additional information is available online in the Supporting Information section at the end of the online article. The authors are solely responsible for the content and functionality of these materials. Queries (other than absence of the material) should be directed to the corresponding author.
