# Supplementary material for: Human impacts on mammals in and around a protected area before, during, and after COVID‐19 lockdowns
Source: Conserv Sci Pract. 2022 Jun 7;4(7):e12743. doi: 10.1111/csp2.12743 (PMC9347595; doi:10.1111/csp2.12743)
Supplement: Supplementary file 1 — APPENDIX S1 Camera activity plot illustrating each individual camera station's periods of activity and inactivity. The x‐axis shows time throughout the entire monitoring period, while the y‐axis indicates specific camera stations. Lines indicate periods where each camera was active, and gaps indicate periods of inactivity [file CSP2-4-0-s005.docx]

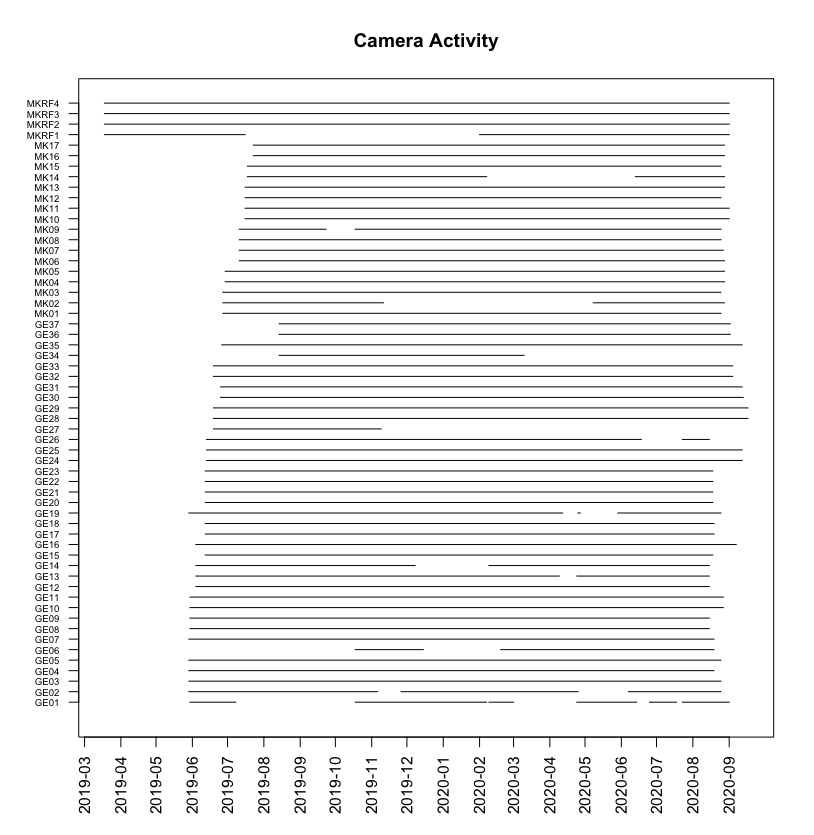


Appendix S1: Camera activity plot illustrating each individual camera station’s periods of activity and inactivity. The x-axis shows time throughout the entire monitoring period, while the y-axis indicates specific camera stations. Lines indicate periods where each camera was active, and gaps indicate periods of inactivity.
