# Supplementary material for: Human impacts on mammals in and around a protected area before, during, and after COVID‐19 lockdowns
Source: Conserv Sci Pract. 2022 Jun 7;4(7):e12743. doi: 10.1111/csp2.12743 (PMC9347595; doi:10.1111/csp2.12743)
Supplement: Supplementary file 2 — APPENDIX S2 Correlations for pairs of predictor variables. Variables are contrasted against each other to illustrate (a) Pearson's rho for all continuous variables, (b) Kendall's tau and (c) Spearman's rho for all variables, but particularly the binomial variable, Trail. All variables had absolute values of correlations < = 0.7. Abbreviated variables are as follows: “Trail” = binary indication (0, 1) of whether the camera was situated along a trail/road (1) or not (0), “Mounted Rec.” = Mounted Recreationists (detection rate), “Stand Ht.” = Stand height (m), “Dist. to Water” = Distance to water (m), “Dist. to bound” = Distance to the urban‐wildland boundary (m), “Pct. Harvested” = Percent of forest harvest (%) in a 500 m buffer around the camera station, “Cam. Ht.” = Camera height (m), “Dist. to Target” = Distance from the camera lens to the expected path of the target (trail, road, or game trail) (m), and “NDVI” = Normalized difference vegetation index extracted from MODIS satellite at 500 m, 8‐day resolution [file CSP2-4-0-s002.docx]

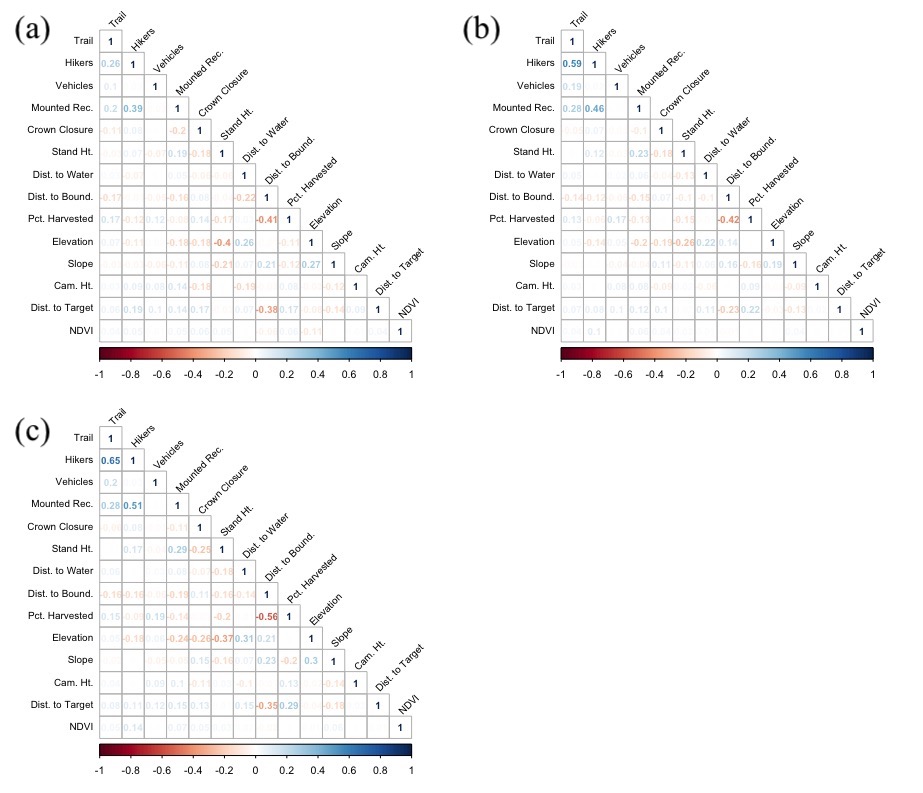


Appendix S2: Correlations for pairs of predictor variables. Variables are contrasted against each other to illustrate (a) Pearson’s rho for all continuous variables, (b) Kendall’s tau and (c) Spearman’s rho for all variables, but particularly the binomial variable, Trail. All variables had absolute values of correlations < = 0.7. Abbreviated variables are as follows: “Trail” = binary indication (0, 1) of whether the camera was situated along a trail/road (1) or not (0), “Mounted Rec.” = Mounted Recreationists (detection rate), “Stand Ht.” = Stand height (m), “Dist. to Water” = Distance to water (m), “Dist. to bound” = Distance to the urban-wildland boundary (m), “Pct. Harvested” = Percent of forest harvest (%) in a 500 m buffer around the camera station, “Cam. Ht.” = Camera height (m), “Dist. to Target” = Distance from the camera lens to the expected path of the target (trail, road, or game trail) (m), and “NDVI” = Normalized difference vegetation index extracted from MODIS satellite at 500 m, 8-day resolution.
