# Supplementary material for: Human impacts on mammals in and around a protected area before, during, and after COVID‐19 lockdowns
Source: Conserv Sci Pract. 2022 Jun 7;4(7):e12743. doi: 10.1111/csp2.12743 (PMC9347595; doi:10.1111/csp2.12743)
Supplement: Supplementary file 3 — APPENDIX S3 (a) Number of independent detections (x‐axis) of each species (y‐axis) based on a 30‐min independence threshold for all mammalian wildlife species detected throughout the study. (b) Proportion of stations (x‐axis) at which each species (y‐axis) was detected throughout the study [file CSP2-4-0-s003.docx]

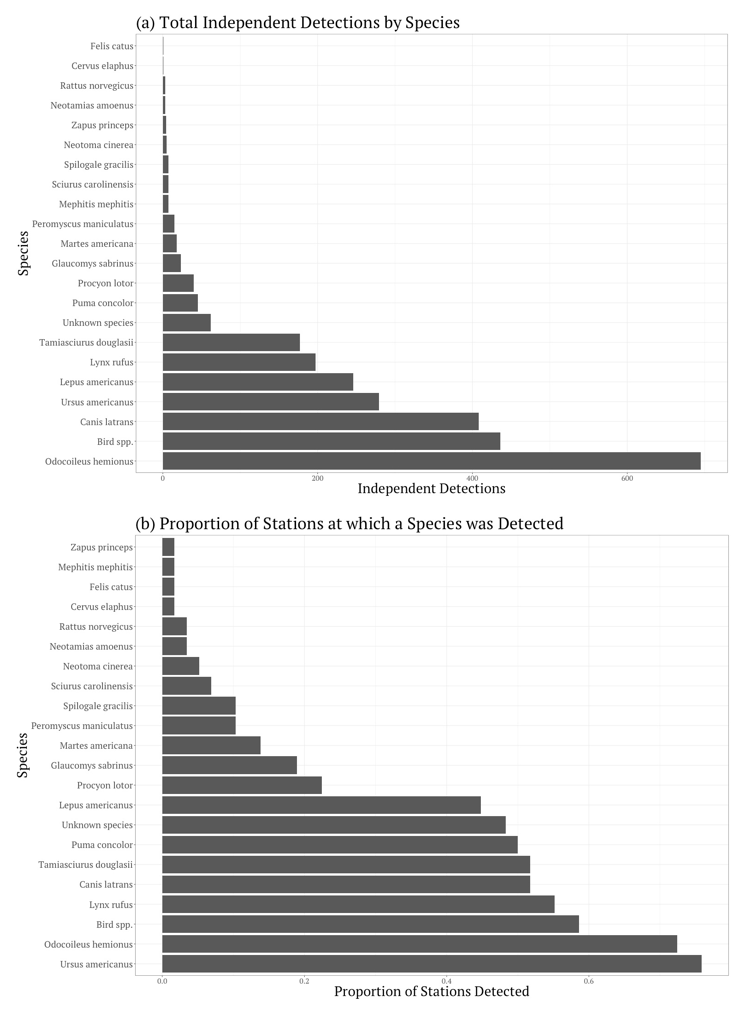
 Appendix S3: (a) Number of independent detections (x-axis) of each species (y-axis) based on a 30-minute independence threshold for all mammalian wildlife species detected throughout the study. (b) Proportion of stations (x-axis) at which each species (y-axis) was detected throughout the study.
