# Supplementary material for: Human impacts on mammals in and around a protected area before, during, and after COVID‐19 lockdowns
Source: Conserv Sci Pract. 2022 Jun 7;4(7):e12743. doi: 10.1111/csp2.12743 (PMC9347595; doi:10.1111/csp2.12743)
Supplement: Supplementary file 4 — APPENDIX S4 Full results from Bayesian generalized linear models for wildlife probability of weekly habitat use against various measures of human activity, while controlling for alternative sources of variation. Bold text indicates predictor variables which had strong effects on weekly wildlife habitat use [file CSP2-4-0-s001.docx]

Appendix S4: Full results from Bayesian generalized linear models for wildlife probability of weekly habitat use against various measures of human activity, while controlling for alternative sources of variation. Bold text indicates predictor variables which had strong effects on weekly wildlife habitat use.

| Species | Variable | Estimate | Lower CI*^a^* | Upper CI*^a^* | R-hat |
| --- | --- | --- | --- | --- | --- |
| *Puma concolor* | Intercept | -6.19 | -7.23 | -5.32 | 1.00 |
|  | Hikers | -0.29 | -0.84 | 0.11 | 1.00 |
|  | Vehicles | -0.22 | -0.91 | 0.13 | 1.00 |
|  | Mounted rec. *^b^* | -0.08 | -0.54 | 0.24 | 1.00 |
|  | Crown closure | 0.38 | -0.05 | 0.88 | 1.00 |
|  | Stand height | 0.29 | -0.13 | 0.71 | 1.00 |
|  | NDVI*^c^* | 0.13 | -0.19 | 0.49 | 1.00 |
|  | Pct. harvested*^d^* | -0.08 | -0.51 | 0.33 | 1.00 |
|  | Dist. to water*^e^* | 0.20 | -0.17 | 0.56 | 1.00 |
|  | Dist. to boundary*^f^* | 0.29 | -0.12 | 0.70 | 1.00 |
|  | **Trail*^g^*** | **2.04** | **1.11** | **3.10** | **1.00** |
|  | Elevation | -0.03 | -0.55 | 0.47 | 1.00 |
|  | Slope | 0.22 | -0.13 | 0.58 | 1.00 |
|  | Cam. ht. *^h^* | 0.28 | -0.10 | 0.66 | 1.00 |
|  | Dist. to target*^i^* | 0.12 | -0.27 | 0.50 | 1.00 |
| *Ursus americanus* | Intercept | -4.30 | -5.01 | -3.67 | 1.00 |
|  | Hikers | -0.25 | -0.62 | 0.06 | 1.00 |
|  | **Vehicles** | **-0.28** | **-0.61** | **-0.05** | **1.00** |
|  | Mounted rec. | 0.15 | -0.03 | 0.32 | 1.00 |
|  | Crown closure | 0.03 | -0.38 | 0.44 | 1.00 |
|  | Stand height | -0.03 | -0.45 | 0.38 | 1.00 |
|  | **NDVI** | **0.66** | **0.41** | **0.93** | **1.00** |
|  | Pct. harvested | 0.25 | -0.16 | 0.69 | 1.00 |
|  | Dist. to water | -0.13 | -0.55 | 0.27 | 1.00 |
|  | Dist. to boundary | -0.23 | -0.71 | 0.23 | 1.00 |
|  | **Trail** | **1.38** | **0.59** | **2.21** | **1.00** |
|  | Elevation | 0.10 | -0.35 | 0.56 | 1.00 |
|  | **Slope** | **-0.47** | **-0.89** | **-0.07** | **1.00** |
|  | Cam. ht. | 0.21 | -0.12 | 0.54 | 1.00 |
|  | Dist. to target | -0.07 | -0.45 | 0.29 | 1.00 |
| *Odocoileus hemionus* | Intercept | -2.72 | -3.62 | -1.88 | 1.00 |
|  | Hikers | -0.14 | -0.67 | 0.32 | 1.00 |
|  | Vehicles | -0.02 | -0.11 | 0.07 | 1.00 |
|  | Mounted rec. | -0.03 | -0.33 | 0.21 | 1.00 |
|  | Crown closure | 0.04 | -0.62 | 0.70 | 1.00 |
|  | Stand height | -0.31 | -0.97 | 0.34 | 1.00 |
|  | **NDVI** | **0.21** | **0.07** | **0.36** | **1.00** |
|  | Pct. harvested | 0.61 | -0.02 | 1.28 | 1.00 |
|  | Dist. to water | -0.19 | -0.82 | 0.42 | 1.00 |
|  | **Dist. to boundary** | **-0.87** | **-1.65** | **-0.12** | **1.00** |
|  | Trail | -0.77 | -2.00 | 0.41 | 1.00 |
|  | Elevation | 0.09 | -0.61 | 0.80 | 1.00 |
|  | Slope | -0.03 | -0.64 | 0.59 | 1.00 |
|  | Cam. ht. | 0.17 | -0.24 | 0.60 | 1.00 |
|  | Dist. to target | 0.33 | -0.20 | 0.89 | 1.00 |
| *Lepus americanus* | Intercept | -5.92 | -7.83 | -4.43 | 1.00 |
|  | Hikers | 0.20 | -0.22 | 0.61 | 1.00 |
|  | Vehicles | 0.02 | -0.11 | 0.14 | 1.00 |
|  | Mounted rec. | -0.50 | -1.30 | 0.13 | 1.00 |
|  | Crown closure | 0.38 | -0.66 | 1.47 | 1.00 |
|  | Stand height | 0.87 | -0.17 | 2.01 | 1.00 |
|  | **NDVI** | **0.28** | **0.06** | **0.53** | **1.00** |
|  | Pct. harvested | 0.46 | -0.47 | 1.50 | 1.00 |
|  | Dist. to water | 0.01 | -0.82 | 0.89 | 1.00 |
|  | **Dist. to boundary** | **-1.82** | **-3.44** | **-0.51** | **1.00** |
|  | Trail | 0.20 | -1.67 | 2.06 | 1.00 |
|  | Elevation | 0.58 | -0.51 | 1.69 | 1.00 |
|  | **Slope** | **-1.02** | **-2.21** | **-0.01** | **1.00** |
|  | Cam. ht. | -0.43 | -1.33 | 0.36 | 1.00 |
|  | Dist. to target | -0.77 | -1.70 | 0.13 | 1.00 |
| *Canis latrans* | Intercept | -6.53 | -8.02 | -5.34 | 1.00 |
|  | Hikers | 0.00 | -0.26 | 0.25 | 1.00 |
|  | Vehicles | 0.03 | -0.09 | 0.13 | 1.00 |
|  | Mounted rec. | 0.01 | -0.14 | 0.15 | 1.00 |
|  | Crown closure | -0.33 | -0.89 | 0.21 | 1.00 |
|  | Stand height | -0.19 | -0.81 | 0.42 | 1.00 |
|  | **NDVI** | **0.28** | **0.06** | **0.53** | **1.00** |
|  | Pct. harvested | 0.25 | -0.29 | 0.82 | 1.00 |
|  | Dist. to water | 0.10 | -0.38 | 0.61 | 1.00 |
|  | **Dist. to boundary** | **-1.57** | **-2.73** | **-0.60** | **1.00** |
|  | **Trail** | **2.69** | **1.46** | **4.11** | **1.00** |
|  | **Elevation** | **-1.11** | **-2.01** | **-0.30** | **1.00** |
|  | Slope | 0.01 | -0.61 | 0.65 | 1.00 |
|  | Cam. ht. | -0.30 | -0.77 | 0.13 | 1.00 |
|  | Dist. to target | 0.30 | -0.18 | 0.81 | 1.00 |
| *Lynx rufus* | Intercept | -6.82 | -8.19 | -5.66 | 1.00 |
|  | **Hikers** | **-0.58** | **-1.09** | **-0.12** | **1.00** |
|  | Vehicles | 0.04 | -0.06 | 0.14 | 1.00 |
|  | Mounted rec. | 0.17 | -0.01 | 0.36 | 1.00 |
|  | Crown closure | 0.43 | -0.12 | 1.00 | 1.00 |
|  | Stand height | 0.44 | -0.12 | 0.98 | 1.00 |
|  | **NDVI** | **0.26** | **0.04** | **0.51** | **1.00** |
|  | Pct. harvested | -0.27 | -0.85 | 0.28 | 1.00 |
|  | Dist. to water | 0.05 | -0.41 | 0.53 | 1.00 |
|  | **Dist. to boundary** | **-1.19** | **-2.09** | **-0.37** | **1.00** |
|  | **Trail** | **3.36** | **2.11** | **4.74** | **1.00** |
|  | Elevation | 0.18 | -0.40 | 0.74 | 1.00 |
|  | Slope | -0.46 | -1.08 | 0.12 | 1.00 |
|  | Cam. ht. | 0.08 | -0.36 | 0.54 | 1.00 |
|  | Dist. to target | 0.16 | -0.33 | 0.65 | 1.00 |

*^a^*Lower CI and Upper CI are the lower and upper 95% credible interval limits for the parameter estimates, respectively.

*^b^*“Mounted Rec.” = Mounted Recreationists (weekly total detections at a camera over the number of days the camera was active that week)

*^c^*“NDVI” = Normalized difference vegetation index extracted from MODIS satellite at 500 m/8-day resolution

*^d^*“Pct. harvested” = Percent of forest harvest (%) in a 500 m buffer around the camera station

*^e^*“Dist. to water” = Distance to water (m)

*^f^*“Dist. to bound” = Distance to the urban-wildland boundary (m)

*^g^*“Trail” = binary indication of whether the camera was situated along a trail/road (1) or not (0)

*^h^*“Cam. ht.” = Camera height (m)

*^i^*“Dist. to target” = Distance from the camera lens to the expected path of the target (trail, road, or game trail) (m).
