# Supplementary material for: Human impacts on mammals in and around a protected area before, during, and after COVID‐19 lockdowns
Source: Conserv Sci Pract. 2022 Jun 7;4(7):e12743. doi: 10.1111/csp2.12743 (PMC9347595; doi:10.1111/csp2.12743)
Supplement: Supplementary file 5 — APPENDIX S5 Results of Bayesian regression models for wildlife detection rates against a categorical designation of time periods before, during, and after the COVID‐19 closures. The baseline intercept was set as the closure period to observe differences between the closure period and before/after. Periods which had strong effects on wildlife detection rates are indicated in bold [file CSP2-4-0-s004.docx]

Appendix S5: Results of Bayesian regression models for wildlife detection rates against a categorical designation of time periods before, during, and after the COVID-19 closures. The baseline intercept was set as the closure period to observe differences between the closure period and before/after. Periods which had strong effects on wildlife detection rates are indicated in bold.

| Species | Area | Period | Estimate | Lower | Upper | R-hat |
| --- | --- | --- | --- | --- | --- | --- |
| *Odocoileus hemionus* | Full | Intercept | 0.028 | 0.013 | 0.044 | 1.00 |
|  |  | Post-Closure | 0.019 | -0.002 | 0.041 | 1.00 |
|  |  | Pre-Closure | 0.002 | -0.019 | 0.024 | 1.00 |
|  | Golden Ears | Intercept | 0.002 | -0.007 | 0.010 | 1.00 |
|  |  | **Post-Closure** | **0.014** | **0.002** | **0.026** | **1.00** |
|  |  | Pre-Closure | 0.008 | -0.004 | 0.020 | 1.00 |
|  | Malcolm Knapp | Intercept | 0.076 | 0.042 | 0.110 | 1.00 |
|  |  | Post-Closure | 0.026 | -0.022 | 0.074 | 1.00 |
|  |  | Pre-Closure | -0.008 | -0.056 | 0.039 | 1.00 |
| *Ursus americanus* | Full | Intercept | 0.018 | 0.011 | 0.025 | 1.00 |
|  |  | Post-Closure | 0.003 | -0.007 | 0.013 | 1.00 |
|  |  | Pre-Closure | 0.000 | -0.010 | 0.010 | 1.00 |
|  | Golden Ears | Intercept | 0.008 | 0.004 | 0.012 | 1.00 |
|  |  | Post-Closure | 0.002 | -0.004 | 0.008 | 1.00 |
|  |  | Pre-Closure | 0.001 | -0.005 | 0.007 | 1.00 |
|  | Malcolm Knapp | Intercept | 0.035 | 0.018 | 0.051 | 1.00 |
|  |  | Post-Closure | 0.006 | -0.018 | 0.029 | 1.00 |
|  |  | Pre-Closure | -0.001 | -0.024 | 0.023 | 1.00 |
| *Canis latrans* | Full | Intercept | 0.021 | 0.007 | 0.035 | 1.00 |
|  |  | Post-Closure | 0.004 | -0.016 | 0.024 | 1.00 |
|  |  | Pre-Closure | -0.002 | -0.023 | 0.018 | 1.00 |
|  | Golden Ears | Intercept | 0.012 | 0.006 | 0.018 | 1.00 |
|  |  | Post-Closure | -0.006 | -0.015 | 0.003 | 1.00 |
|  |  | Pre-Closure | -0.006 | -0.015 | 0.002 | 1.00 |
|  | Malcolm Knapp | Intercept | 0.036 | -0.001 | 0.074 | 1.00 |
|  |  | Post-Closure | 0.020 | -0.031 | 0.072 | 1.00 |
|  |  | Pre-Closure | 0.004 | -0.048 | 0.056 | 1.00 |
| *Lepus americanus* | Full | Intercept | 0.008 | 0.000 | 0.016 | 1.00 |
|  |  | Post-Closure | -0.002 | -0.013 | 0.009 | 1.00 |
|  |  | Pre-Closure | 0.008 | -0.003 | 0.019 | 1.00 |
|  | Golden Ears | Intercept | 0.004 | -0.001 | 0.008 | 1.00 |
|  |  | Post-Closure | -0.001 | -0.007 | 0.005 | 1.00 |
|  |  | Pre-Closure | 0.001 | -0.005 | 0.008 | 1.00 |
|  | Malcolm Knapp | Intercept | 0.015 | -0.004 | 0.035 | 1.00 |
|  |  | Post-Closure | -0.003 | -0.031 | 0.024 | 1.00 |
|  |  | Pre-Closure | 0.020 | -0.007 | 0.048 | 1.00 |
| *Puma concolor* | Full | Intercept | 0.005 | 0.004 | 0.007 | 1.00 |
|  |  | **Post-Closure** | **-0.004** | **-0.006** | **-0.002** | **1.00** |
|  |  | **Pre-Closure** | **-0.005** | **-0.007** | **-0.003** | **1.00** |
|  | Golden Ears | Intercept | 0.007 | 0.005 | 0.009 | 1.00 |
|  |  | **Post-Closure** | **-0.006** | **-0.009** | **-0.003** | **1.00** |
|  |  | **Pre-Closure** | **-0.006** | **-0.009** | **-0.003** | **1.00** |
|  | Malcolm Knapp | Intercept | 0.002 | 0.001 | 0.003 | 1.00 |
|  |  | Post-Closure | 0.000 | -0.002 | 0.002 | 1.00 |
|  |  | Pre-Closure | -0.002 | -0.004 | 0.000 | 1.00 |
| *Lynx rufus* | Full | Intercept | 0.013 | 0.007 | 0.018 | 1.00 |
|  |  | Post-Closure | -0.005 | -0.012 | 0.003 | 1.00 |
|  |  | Pre-Closure | -0.003 | -0.010 | 0.005 | 1.00 |
|  | Golden Ears | Intercept | 0.008 | 0.003 | 0.013 | 1.00 |
|  |  | Post-Closure | -0.003 | -0.010 | 0.004 | 1.00 |
|  |  | Pre-Closure | 0.000 | -0.007 | 0.007 | 1.00 |
|  | Malcolm Knapp | Intercept | 0.022 | 0.010 | 0.034 | 1.00 |
|  |  | Post-Closure | -0.008 | -0.025 | 0.008 | 1.00 |
|  |  | Pre-Closure | -0.008 | -0.025 | 0.009 | 1.00 |
